# MAAMD: a workflow to standardize meta-analyses and comparison of affymetrix microarray data

**DOI:** 10.1186/1471-2105-15-69

**Published:** 2014-03-12

**Authors:** Zhuohui Gan, Jianwu Wang, Nathan Salomonis, Jennifer C Stowe, Gabriel G Haddad, Andrew D McCulloch, Ilkay Altintas, Alexander C Zambon

**Affiliations:** 1 Department of Bioengineering, University of California, San Diego, La Jolla, CA, USA; 2 San Diego Supercomputer Center, University of California, San Diego, La Jolla, CA, USA; 3 Cincinnati Children’s Hospital Research Foundation, Cincinnati, OH, USA; 4 Department of Pediatrics, University of California, San Diego, La Jolla, CA, USA; 5 Department of Pharmacology, University of California, San Diego, La Jolla, CA, USA

**Keywords:** Workflow, Affymetrix microarray, Kepler, AltAnalyze, Meta-analysis, Gene orthologues, Hypoxia

## Abstract

**Background:**

Mandatory deposit of raw microarray data files for public access, prior to study publication, provides significant opportunities to conduct new bioinformatics analyses within and across multiple datasets. Analysis of raw microarray data files (e.g. Affymetrix CEL files) can be time consuming, complex, and requires fundamental computational and bioinformatics skills. The development of analytical workflows to automate these tasks simplifies the processing of, improves the efficiency of, and serves to standardize multiple and sequential analyses. Once installed, workflows facilitate the tedious steps required to run rapid intra- and inter-dataset comparisons.

**Results:**

We developed a workflow to facilitate and standardize **M**eta-**A**nalysis of **A**ffymetrix **M**icroarray **D**ata analysis (MAAMD) in Kepler. Two freely available stand-alone software tools, R and AltAnalyze were embedded in MAAMD. The inputs of MAAMD are user-editable csv files, which contain sample information and parameters describing the locations of input files and required tools. MAAMD was tested by analyzing 4 different GEO datasets from mice and drosophila.

MAAMD automates data downloading, data organization, data quality control assesment, differential gene expression analysis, clustering analysis, pathway visualization, gene-set enrichment analysis, and cross-species orthologous-gene comparisons. MAAMD was utilized to identify gene orthologues responding to hypoxia or hyperoxia in both mice and drosophila. The entire set of analyses for 4 datasets (34 total microarrays) finished in ~ one hour.

**Conclusions:**

MAAMD saves time, minimizes the required computer skills, and offers a standardized procedure for users to analyze microarray datasets and make new intra- and inter-dataset comparisons.

## Background

DNA microarrays were first developed to simultaneously quantify the expression of large numbers of genes. They are now commonly used for a variety of genetic analyses such as alternative splicing, microRNA regulation, and SNP detection. Many DNA microarray platforms have emerged but their fundamental design has remained relatively standard; they consist of arrayed DNA oligonucleotides (known as probes) that are complementary to specific DNA sequences [1]. Since the majority of biomedical journals require that raw and normalized microarray data be accessible to the public at the time of publication, a significant number of datasets are publically available. Turning this huge trove of accessible data into useful conclusions is becoming increasingly problematic for scientists.

Currently, several types of microarray instrument platforms and their associated data formats are available, including: Illumina, Affymetrix, and Agilent. These datasets, once quality controlled and normalized, provide significant opportunities for conducting novel intra- and inter-dataset comparisons. Though only 872 of the roughly 11,000 microarray platforms currently found in the NCBI gene expression omnibus (GEO), are Affymetrix gene chips. Affymetrix platforms currently comprise ~17,300 among total ~38,700 datasets (series). This provides oppportunities to conduct meta-analyses minimizing platform biases from combining data from different array platforms, which can complicate meta-analyses [2].

Gene-set enrichment analysis (GSE), such as gene ontology and pathway enrichment, is a common method for microarray data analysis [3,4]. Improved functional annotations of multiple genomes, increasing numbers of experimentally determined protein-protein interactions and improved pathway level relationships have dramatically increased the quality and scope of results from GSE analyses. Reanalysis of old datasets with the updated gene-set enrichment tools such as Gene Ontology and the Molecular Signatures Database (MSigDB) is likely to identify new signaling networks associated with the analyzed dataset [5]. However, the analysis of microarray data involves multiple steps: data organization, quality control, normalization, differential gene expression calling, clustering and pathway analysis. Therefore, significant bioinformatics skills are required to properly conduct such microarray analyses. The development of a software-based workflow to automate these procedures would improve the efficiency and serve to standardize the multiple inter- and intra-dataset analyses.

Advances in computational techniques have also increased the number of open-source bioinformatics analysis tools for genomic analysis. Gricia et al. developed a workflow generator for microarray and sequence data [6]. Pelizzola et al. developed an R-based package to automate microarray analysis [7]. More R-based bioconductor packages such as GEOquery are now available, however running packages in R requires, for the most part, comfort in command-line interface since GUIs are not available for most R packages. Salomonis et al. developed an open source Python-based software called AltAnalyze for microarray and RNA sequencing analysis with the integrated gene-set enrichment software GO-Elite [8]. AltAnalyze can also be run from a user-friendly GUI or compiled binaries can be run with simple command lines. These features combined with the increasing number of analysis options available in AltAnalyze facilitate its use by both general bench biologists and bioinformaticists wishing to incorporate its many I/O functions into analysis pipelines.

Scientific workflow systems provide an environment to aid the scientific discovery process through the combination of available tools for scientific data management, analysis, simulation, and visualization. Moreover, they provide a comfortable and intuitive graphical interface for designing and modifying workflows [9]. Kepler is open-source software that supports user-customized scientific workflows. Stropps et al. has developed an automated workflow in Kepler utilizing Pelizzola’s package for Affymetrix microarray data analysis [10]. Stropps’ workflow conducts the normalization, differentially expressed gene analysis (DEG), clustering analysis, and gene ontology statistics for one Affymetrix data set. In this study, we sought to develop a scientific workflow to facilitate and standardize the meta-analysis of multiple Affymetrix microarray datasets, regardless of species, utilizing state-of-the-art open-source bioinformatics programs: AltAnalyze and R/bioconductor packages. MAAMD is designed to encapsulate all steps involved in analyzing raw Affymetrix data files available in the GEO repository. MAAMD automates multiple dataset downloading, data organization, data quality control as well as normalization, several statistical methods for differential gene expression determination, mutltiple testing adjustments, clustering, and GO-Elite pathway and gene set enrichment analysis into a single, easy to use workflow. MAAMD is then expanded by enabling across-experiment/species comparisons extending the traditional intra-dataset differential analysis to rapid inter-dataset comparisons.

**Figure 1:**
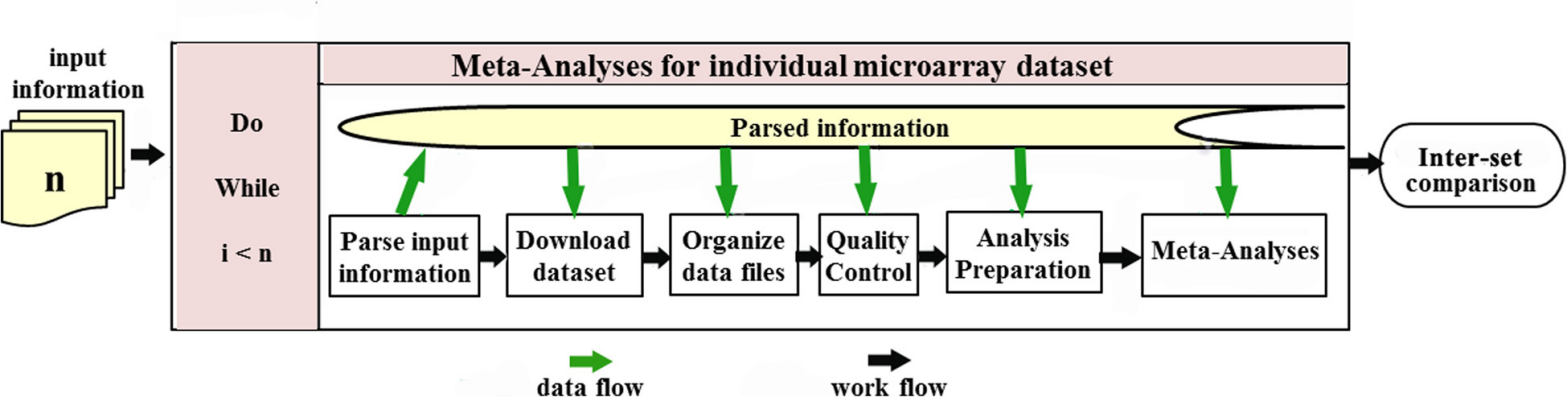
**The conceptual view of MAAMD.** Briefly, each dataset among n targeted datasets is analyzed in a loop one by one. Input files are prepared in advance. MAAMD extracts required information from these input files. The targeted dataset is then downloaded and re-organized. A quality control is executed to assist the users to evaluate the sample quality. MAAMD then prepares for meta-analyses by asking the users to select groups, intra-set comparisons. The meta-analyses are then executed and the results are stored at the assigned location. When all targeted datasets have been analyzed, an inter-set comparison can be executed to identify the common regulated genes among datasets.

Figure 1 shows the conceptual view of MAAMD. Briefly, the targeted datasets were selected from the online database and the corresponding information was collected and input into excel files. The excel files were then parsed and the targeted datasets were downloaded to a local computer and organized locally according to the parsed information. The data quality was analyzed and then assessed by the user. Sample groups for DEG analysis were assigned. The selected data were then normalized and DEG was conducted alongside clustering and gene ontology analyses. The targeted datasets were analyzed individually. After all datasets had been analyzed, the users were allowed to select datasets to conduct a comparison of the differentially-expressed genes across datasets and species using a homologue database incorporated in MAAMD. MAAMD implements the above conceptual workflow in Kepler and simplifies user’s actions by only requiring modification of local excel files and selecting listed options. The function of across-experiment/species comparison in MAAMD expands the traditional intra-dataset differential analysis to inter-dataset comparisons. The utilization of MAAMD was validated by an across-species hypoxia and hyperoxia dataset comparisons.

## Implementation

### Software components of MAAMD

Kepler is a free, open-source software system for designing, executing, reusing, evolving, archiving, and sharing scientific workflows [10,11]. It has been widely used in many areas of science that require sequential and parallel manipulation of large and complex datasets [10]. The free availability, the capacity of multiplatform development, and the capability of utilizing existing packages or tools developed by R and Python make Kepler ideal for academic applications. In addition, Kepler presents workflows in a graphical format which promotes rapid comprehension of inputs, data flow and data processing [10]. All components in Kepler are easily customizable, which leads to a fast and efficient extension and modification of the Kepler workflow. A new platform, “bioKepler”, developed for bioinformatics, will be available soon (bioKepler.org). Decades of available bioinformatics tools will be embedded in bioKepler and hence simplify the development and scalability of scientific workflows for bioinformatics in Kepler.

AltAnalyze is a Python-based open-source, cross-platform, free application for microarray and RNA sequencing analysis [12]. It is packaged with an intuitive easy to use GUI, but has also been compiled on various platforms to enable users to run it in command line. AltAnalyze is a multi-functional software package that can summarize, organize and filter exon and junction-level data, calculate statistical scores for alternative splicing, alternative promoter selection or alternative 3′end processing, annotate regulated alternative exon events, and assess downstream predicted functional consequences at the level of protein domains, microRNA binding sites, and biological pathways (http://altanalyze.org). AltAnalyze has its own up-to-date relational database structure that stores the information of platforms, probesets, and species. Affymetrix library and annotation files are automatically identified and downloaded by AltAnalyze for a large variety of existing Affymetrix array platforms and species. AltAnalyze can also conduct data normalization and pathway analyses for a variety of other platforms (e.g. high content RNA sequencing, Illumina and Agilent). This software is also compatible with user-provided Affymetrix library files and supports the processing of non-Affymetrix normalized or raw tab-delimited expression files, including proteomics and metabolomics data.

The data resulting from running AltAnalyze include a series of text files that can be easily opened and further analyzed with standard spreadsheet programs. Graphical QC plots, hierarchical clustering heat maps, Principle Component Analysis (PCA) and pathway diagrams can be produced using the operating system-specific compiled versions of the source code. A large set of ontologies and other gene sets, including transcription factor targets, are available for GSE from AltAnalyze via the integrated GO-Elite analysis tool [8]. The latest version of AltAnalyze incorporates a multi-threading technique, which improves the processing speed. With these rich features, AltAnalyze can conduct an analysis of microarray data comprehensively and efficiently [12,13], even for those new to such analyses.

### The MAAMD workflow

**Figure 2:**
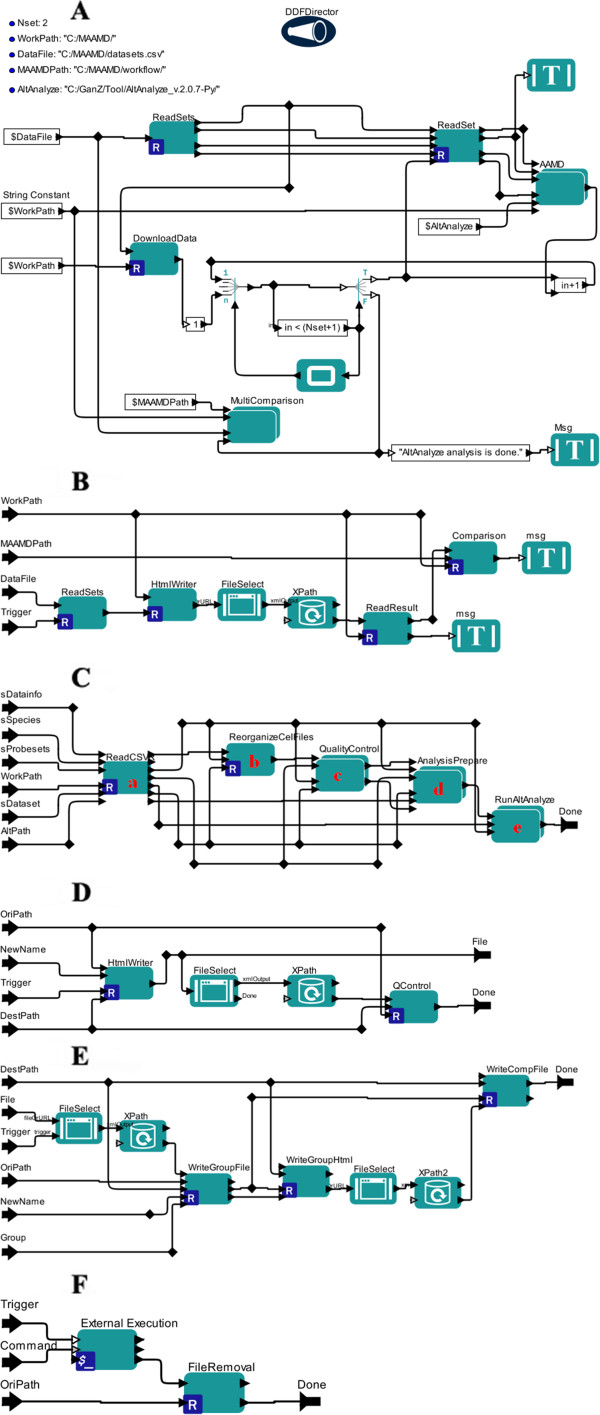
**The implementation of MAAMD in Kepler. (A)** Design of the entire workflow. **(B)** Design of module e in Figure 2A. **(C)** Design of module d in Figure 2A. **(D)** Design of module c in Figure 2C. **(E)** Design of module d in Figure 2C. **(F)** Design of module e in Figure 2C.

As designed, MAAMD has functions such as downloading available online data, re-organizing data, estimating data quality, grouping data, conducting a differential gene-expression analysis, and inter-dataset comparisons. The implementation of MAAMD is shown in Figure 2. The workflow was represented as a network of actors, which were the basic components for task execution. It was executed by a director, which was used to manage and schedule the execution of actors. The data was delivered between the actors with the connections between basic actors and the input/output ports in comprehensive actors.

### A. Kepler workflow

Briefly, this workflow consists of one DDF director, five customized actors, one loop control, and two display actors. Five string inputs are required: the number of targeted datasets, the location of the MAAMD workflow file, the location of the AltAnalyze executable file, the working directory, and the location of the file containing information for targeted datasets. The output is a folder with a fixed structure containing original data files, meta-analyses results by AltAnalyze, and the comparison results across the datasets. Once initiated, minimal user-interactions are required to complete the workflow.

Figure 2 indicates the detailed implementation of MAAMD workflow. The input information for the targeted datasets was parsed by module A, followed by the downloading and uncompressing of targeted data in module B. Then the information of individual datasets was parsed one by one in module C, controlled by an index control loop. The parsed information of an individual dataset was delivered to a composite actor module D, which conducts data quality estimation, data selection, normalization, and analyses. When the analyses of all datasets finished, a comparison among the results of these datasets was then executed in module E.

Among these modules, module D (Figure 2C), which analyzes individual datasets, is a central module. This module is a composite Kepler actor containing five basic actors. Briefly, the sample information in a single dataset was parsed in module A. The data was re-organized to facilitate the data identification and operation in module B. The data quality was then estimated by a composite actor, module C, whose structure was represented in Figure 2D. Based on the quality report, the user selects the qualified data in module D. The selected data was then normalized and analyzed by AltAnalyze in module E. When module E finishes its processing, it sends out a “Done” signal to trigger the next step.

Here are the brief introductions of each module in MAAMD.

#### ***Module A: ReadSets***

The module “ReadSets” is the first module of MAAMD that is used to parse the dataset information contained in “datasets.csv” file. This module is an R-based Kepler actor. The input of this module is a string parameter that describes the location of csv file which is the parameter “DataFile” in Figure 2A. The output is the parsed information.

#### ***Module B: DownloadData***

The module “DownloadData” is also an R-based Kepler actor that downloads and decompresses targeted datasets. The inputs of DownloadData are the parsed information from Module A and the path of work directory. Its output is the uncompressed.CEL files that are stored in a folder named as the assigned “Dataset” value in the work directory assigned by the parameter “WorkPath”.

#### ***Module C: ReadSet***

The module “ReadSet” is designed to get the information for one dataset record, controlled by an index loop. The inputs of ReadSet are the parsed total information for all datasets listed in “datasets.csv” from Module A and a trigger signal from Module B. The output of ReadSet is the parsed information for one dataset.

#### ***Module D: AAMD***

The module “AAMD” is a composite actor containing 5 child actors. The inputs of AAMD are the parsed information from Module B, the location of AltAnalyze, and the working directory. The output of AAMD is a “Done” signal and a folder containing analyzed results. The following are the child actors involved in AAMD.

#### ***ReadCSV***

The actor “ReadCSV” reads information of samples in the targeted dataset including the original sample names and the corresponding customized names as well as group information. An AltAnalyze command is generated based on the input information. The inputs of ReadCSV are all inputs of AAMD module. The outputs are the parsed sample information and an AltAnalyze command.

#### ***ReorganizeCelFiles***

The actor “ReorganizeCelFiles” renames the CEL files with its corresponding customized names. The inputs are the old file names, the new file names, and the location of files. The output of ReorganizeCelFiles is a “Done” signal.

#### ***QualityControl***

**Figure 3:**
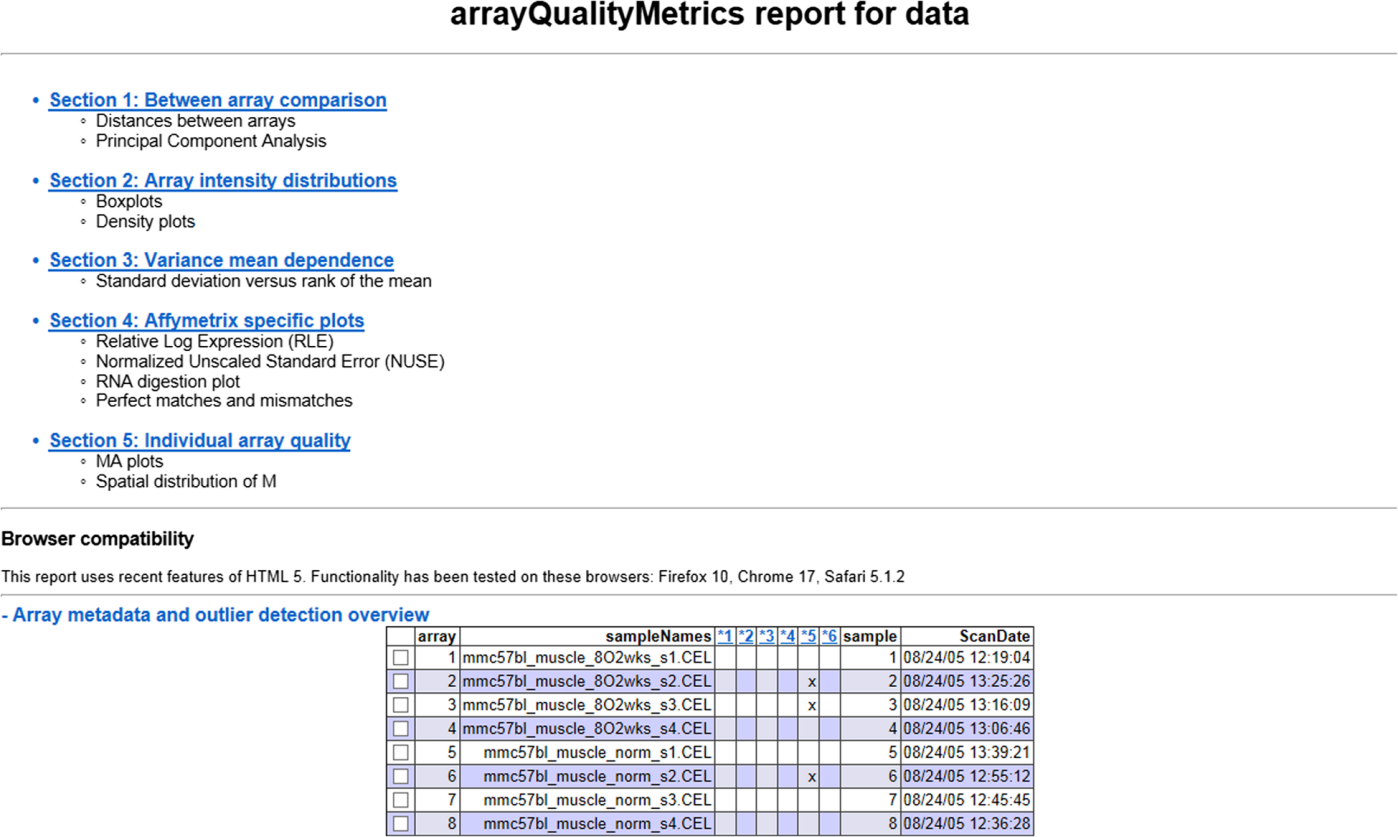
**A screenshot of the summary section of the quality control report for dataset GSE9400.** The quality control report includes five sections “Between array comparison”, “Array intensity distribution”, “Variance mean dependence”, “Affymetrix specific plots” and “Individual array quality”.

“QualityControl” is a composite actor that consists of four basic actors. The detailed structure of QualityControl is shown in Figure 2D. This actor allows the user to select CEL files and estimate the quality of the selected data. The Bioconductor packages “*arrayQualityMetrics”* and “*affyQCReport*” were utilized through R. The inputs of QualityControl are a trigger signal, the location of sample data, the folder to store results, and the customized sample names. A file named “celllist.html” is created. The quality report is saved as a webpage. Figure 3 shows the summary section of the resulting webpage, which includes array intensity analysis, principle component analysis, distance analysis and so on.

#### ***AnalysisPrepare***

“AnalysisPrepare” allows the users to select CEL files for further analysis based on the quality control report. The actor creates a “groups.txt” file describing group information and a “comps.txt” file describing the selected comparisons for further AltAnalyze analysis. A web page listing all possible comparisons between groups is created to allow users to select specific group comparisons. The inputs of AnalysisPrepare are parsed sample information and a trigger signal. The output is a “Done” signal.

#### ***RunAltAnalyze***

“RunAltAnalyze” calls AltAnalyze by a command line and then analyzes the data in AltAnalyze. The inputs of RunAltAnalyze are the command line and the location of selected data. A “result” folder is created to store the analysis results in the folder named by its corresponding dataset name. The output is a “Done” signal.

#### ***Module E. MultiComparison***

The module “MultiComparison” is a composite actor to compare the differential gene expression among datasets across experimental conditions or species. The detailed structure of MultiComparison is shown in Figure 2B. This module is independent and can run without other modules as long as AltAnalyze has performed the analyses of targeted datasets. MultiComparison collects the analyzed results of targeted datasets and converts them into orthologous genes. The orthologous genes existing in all targeted datasets are summarized in a “ComparisonSets.txt” file. The inputs of MultiComparison are “datasets.csv”, the location of analyzed results, and the location of MAAMD workflows. A Kepler display pops up at the end of the analyses to show the summary of the comparison results or to remind the user if no gene orthologue can be found across multiple datasets.

### B. Input of MAAMD

The inputs of MAAMD workflow are five string parameters as showed in Figure 2A. The parameter “DataFile” assigns the location of the file containing the information of targeted datasets. Table 1 is an example of the related file. Briefly, this file is a csv file containing four columns: “Dataset”, “Species”, “Probesets” and “Datainfo”. The columns in Table 1 are fixed, but the user is allowed to edit the table content. The column “Dataset” describes the name of targeted dataset. “Species” and “Probesets” describe the corresponding animal species and the probesets used to acquire the data, respectively. “Datainfo” is a path linking to a csv file that describes the samples in the individual dataset. Table 2 is an example of a csv file assigned by “Datainfo”. “Datainfo” contains three columns: “SampleName”, “NewName”, and “Group”. “SampleName” describes the original sample names of the downloaded dataset. “NewName” is a customized file name containing proper information such as animal species, tissue, and experimental conditions to identify samples. “Group” assigns samples to a specific group. At least two groups are expected in one dataset. Thus, beside the five string parameters, MAAMD also requires a datasets csv file along with corresponding csv files for each dataset.

**Table 1:** An example of the csv file to describe targeted datasets

**Dataset**	**Species**	**Probesets**	**Datainfo**
gse15879	Dm	Drosophila_2	C:/MAAMD/datainfo-gse15879.csv
gse14981	Dm	Drosophila_2	C:/MAAMD/datainfo-gse14981.csv
gse12160	Dm	Drosophila_2	C:/MAAMD/datainfo-gse12160.csv
gse9400	Mm	Mouse430_2	C:/MAAMD/datainfo-gse9400.csv

Listed in Table 1 is the content of “datasets.csv” that describes the targeted datasets for the study case.

**Table 2:** An example of the csv file to describe the samples in one dataset

**Sample name**	**New name**	**Group**
GSM239142.CEL	mmc57bl_muscle_norm_s1.CEL	con
GSM239143.CEL	mmc57bl_muscle_norm_s2.CEL	con
GSM239144.CEL	mmc57bl_muscle_norm_s3.CEL	con
GSM239145.CEL	mmc57bl_muscle_norm_s4.CEL	con
GSM239146.CEL	mmc57bl_muscle_802wks_s1.CEL	hyp
GSM239147.CEL	mmc57bl_muscle_802wks_s2.CEL	hyp
GSM239148.CEL	mmc57bl_muscle_802wks_s3.CEL	hyp
GSM239149.CEL	mmc57bl_muscle_802wks_s4.CEL	hyp

Listed in Table 2 is the content of “datainfo-gse9400.csv” that supplies information for the samples in GSE9400 dataset.

### C. The work and output folders of MAAMD

**Figure 4:**
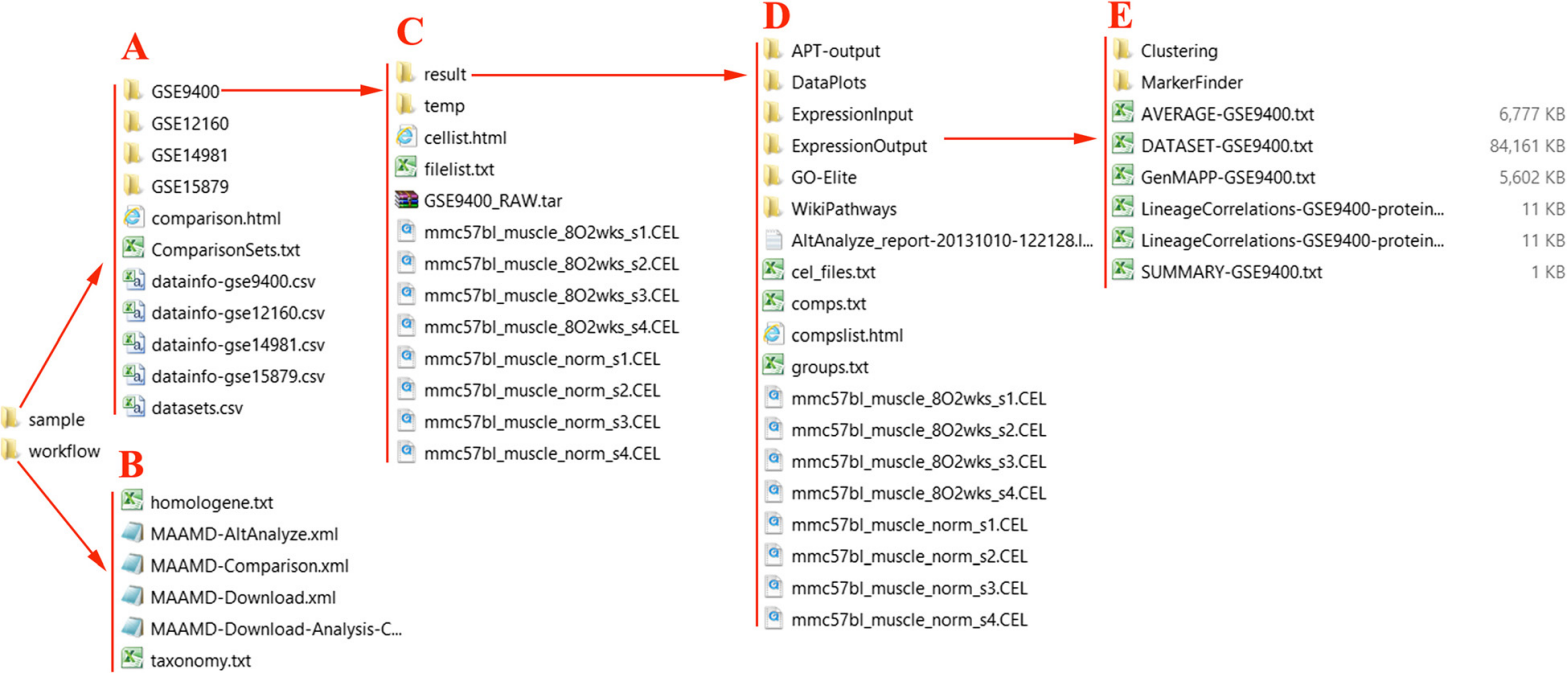
**The structure of work and output folder of MAAMD. (A)** Contents of the work folder. **(B)** the concent of “workflow” floder. **(C)** the concent of the output folder for an individual data set “GSE9400”. **(D)** the concent of “result” folder for GSE9400. **(E)** the content of “ExpressionOutput” folder in the “result” folder of GSE9400 where “DATASET-GSE9400.txt” is located.

The output folder of MAAMD is a folder assigned by “WorkPath”. Figure 4 is an example of work and output folders. Figure 4A shows the content of the result folder where, “GSE9400”, “GSE12160”, “GSE14981” and “GSE15879” store the original data and analysis results for corresponding datasets. A text file “ComparisonSets.txt” contains orthologues between datasets. Figure 4B shows the content of “workflow” folder which contains the Kepler workflow files and required resource files “homologene.txt” and “taxonomy.txt”. Figure 4C shows the detailed structure of folder “GSE9400”. The detailed structure of the “result” folder is shown in Figure 4D. The differential gene expression results with statisical evaluation are summarized in a file named “DATASET-GSE9400.txt” in the “ExpressionOutput” folder as shown in Figure 4E. More details about the outputs of AltAnalyze are available on the AltAnalyze’s website http://www.altanalyze.org/help_main.htm.

### D. Software requirements of MAAMD workflow

The following software programs are required to run MAAMD: Kepler 2.4 or higher; Java run-time environment (which is a Kepler dependency); R 3.0.0 to support the R actors in Kepler; AltAnalyze 2.0.8 or higher. When running the source version of AltAnalyze, rather than compiled, additional dependencies are required as described here: http://code.google.com/p/altanalyze/wiki/StandAloneDependencies. Several Bioconductor R packages including *GEOquery*, *AffyQCReport*, and *arrayQualityMetrics* must be installed in advance. Detailed instructions with illustrations for how to set up a computer environment to run MAAMD are described in Supplement A.

A minimum of 1GB of RAM and at least a 2GHz CPU are required to run this workflow. The required disk space for the workflow outputs depends on the size of selected targeted datasets. Additional RAM (up to 8GB) and hard-drive space (up to 3GB free) are recommended for large microarray datasets or splicing-sensitive platform studies.

### E. A case study of MAAMD performance: preliminary analysis for a comparative hypoxia study

We conducted a case study of MAAMD to test the speed at which it was able to download, qualtity control, analyze and compare multiple expermiments across diverse species (mouse and Drosophila). Hypoxia, exposure of cells and tissues to O_2_ levels below ~5%, is widely associated with many diseases and its effects contribute to millions of clinical cases every year in the United States [14,15]. The underlying conserved cellular responses to hypoxia are poorly understood. A study of orthologues responding to different hypoxic conditions or in different species could identify potential conserved genes and help further understand conserved cellular adaptations to hypoxia. Thus, we used MAAMD to identify transcriptional responses to hypoxia that are conserved in multiple hypoxia experiments and between mice and Drosophila.

Affymetrix Microarray data was downloaded from the NCBI Gene Expression Omnibus (GEO), a publicly available functional genomics data repository. We selected the Affymetrix datasets *GSE9400*, *GSE12160*[16], *GSE14981*[17], and *GSE15879*[18] consisting of profiling data from flies or mice exposed to a range of hypoxic or hyperoxic conditions. Specifics about each of these datasets can be found at the GEO website.

A summary csv file “datasets.csv” and four csv files for individual datasets “datainfo-gse9400.csv”, “datainfo-gse14981.csv”, “datainfo-gse15879.csv”, and “datainfo-gse12160.csv” were prepared based on the experimental description by the user as the input files of MAAMD. The input parameters were set as the locations of input files, AltAnalyze, MAAMD, and the total number of targeted datasets. Then, MAAMD was executed using a computer configured with Intel i7-3517U CPU 1.9 GHz, 8 GB RAM, 120 G disk space, and a Windows 8 64 bit OS.

The entire process, including computer analysis and user operation, took ~45 min to analyze all four datasets. The analysis, including data downloading and data meta-analysis took MAAMD a total of ~41 min. Among these processes, the data downloading took ~2 minutes with the high-speed campus internet; the meta-analysis for all four datasets including data quality estimation, differential gene expression, and GO-Elite analysis took ~37 minutes; the cross-species comparison of results for all four datasets took ~2 minutes (Table 3). The requird time may vary due to the differences in hardware configuration or internet speed. Conducting this analyisis independently and by hand could take upwards of 4–5 hours for an experienced user implimenting the exact same set of independent programs. This time would be spent providing repeated user inputs to create and navigate folders, check results, rename files, select criteria, formatting I/O files and so on.

**Table 3:** The list of common regulated genes between datasets

**Compared dataset**	**Significance**	**Common regulated genes**		
		**Total**	**Conserved genes**	**Differential genes**
GSE15879 v.s. GSE14981	A comparison between chronic and acute hypoxia in flies	7	Hsp26, HSPA1A, GstD1, CG14120	CG3734, CG13607, Cyp4d2
GSE15879 v.s. GSE12160	A comparison between chronic hypoxia and chronic hyperoxia in flies	12	Hsp26, CG14120, RGN, CG31300, LOC423786	Lsp1beta, CG15766, Cyp4d2, CG5897, HPGD, Lsd-1, si:dkey-7814.10
GSE15879 v.s. GSE9400	A comparison of chronic hypoxia in mice and flies	4	RRM2, AGPAT3	RRM1, FBP2
GSE15879 v.s. GSE14981 v.s. GSE12160	A comparison of chronic hypoxia, chronic hyperoxia and acute hypoxia in flies	3	Hsp26, CG14120	Cyp4d2

The column “Compared Datasets” lists the detailed comparisons. The column “Significance” states the biological meaning to make such a comparison. The column “Common Regulated Genes” lists the total number of common regulated genes, the detailed conserved genes and the detailed differential genes.

MAAMD generated about 850 Mb of results including the downloaded data for above four datasets. Visualizations such as heatmaps for clustering analysis, pathway maps for pathway analysis were generated based on the resulting values automatically. The detailed values along with statistical evaluation were stored in files in “ExpressionOutput” folder and “GO-Elite” folder of each dataset. Moreover, we were able to make multiple comparisions across all datasets quickly and automatically.

The results indicate highly conserved gene responses to whole-animal exposure to hypoxia or hyperoxia in all datasets. GSE9400 contained profiles of soleus muscles from C57BL/10 mice exposed to gradient 8% O_2_ for 2 weeks revealed 2293 differentially expressed probesets (>2 fold change up or down, p < 0.05). GSE15879 contained profiles of flies exposed to a decreasing levels of hypoxia for 16 days displayed 201 probesets using the same fold and P-value cutoffs. In the two drosophila datasets, 28 probesets were signficantly changed in GSE14981 (flies exposed to 1% O_2_ for 2.5 hours) and 109 probesets were changed in GSE12160 (hyperoxia-selected flies).

Given the related yet significantly different experimental conditions, inter-set comparisons were still able to identify conserved gene expression responses between different inter-set comparisons. Table 3 summarizes the comparisons made and corresponding findings. Based on the results, genes Hsp26 and CG14120 are conserved in responding to chronic hypoxia, acute hypoxia and chronic hyperoxia in drosophila. This result is consistent with the knowledge that heat shock proteins encoding Hsp genes are conserved proteins responding to diverse environmental stresses [19]. Little is known about CG14120, suggesting that using this meta-analyis in MAAMD can identify novel conserved functionally-uncharacterized genes that are regulated by divergent yet related (hypoxia vs hyperoxia) stress conditions. Depending on the comparisons made, MAAMD was able to rapidly identify subsets of genes that were regulated both in the same directions and in opposite directions across different hypoxic stresses, tissues and species very quickly (Table 3).

In summary, these results highlight the orthologue comparison function of MAAMD, the dramatic savings in time to properly conduct a cross-species meta-analysis and identify highly conserved gene responses.

## Results and discussion

The “MAAMD” workflow was developed and demonstrated to work efficiently for Affymetrix microarray data, the predominant plaftorm currently represented in GEO. Because of its design in Kepler, it can be easily adapted to analyze other platforms. This workflow improves the effeciency of microarray analysis and significantly minimizes the required computational and bioinformatic knowledge for the user, especially once installed. The extended function, which compares the analyzed results from multiple microarray datasets across species, further simplifies the work required by the user and significantly broadens its utility.

MAAMD improves the analysis efficiency by linking together previously separate tasks of data flow and avoiding the repeated memory or CPU resource allocation since the resources are assigned once it starts to run. Furthermore, the embedded tools such as AltAnalyze, R-based comparison codes are efficient in data processing. For example, the cross-species analysis between the datasets took only 3 minutes, which could be a very complicated and time-consuming process. The factors which affect MAAMD running time include the size of dataset, the type of species being analyzed and internet speed. The size of the dataset being downloaded and analyzed has a large effect on workflow execution time at almost every step from data downloading to data quality control and data analysis. The speed of the internet connection can significantly impact the time required for data downloading considering a single Affymetrix CEL file is on average ~10 MB in size. The species analyzed will also have an impact on the performance time, as a result of genome complexity and degree of annotation.

As an automated workflow, MAAMD avoids random operational mistakes such as a misclassfication of data files or erroroneous group comparisons due to simple human error. MAAMD minimizes the required user interaction by only occassionally pausing to recieve user inputs. To improve the flexibility and feasbility of MAAMD, there are several user interactions included in this workflow, such as the selection of data files before and after quality control. Some microarray datasets contain CEL files acquired by different platforms. This usually causes errors if a user analyzes them as a single batch. Data selection for further analysis after sample QC and proper selection of similar control types between datasets should avoid these kinds of errors. All of the required user interactions appear to the users as a web page with listed options which are direct and easy to understand and control.

The inputs of MAAMD are csv files summarizing the information of targeted datasets. Since the inputs are text-based tables and selectable options in lists, use of MAAMD does not require advanced computational skills. Additionally, MAAMD provides a standard and easily modifyable workflow for genetic data analysis such as RMA normalization, differential expression analysis, and clustering analysis with its built-in tools, Bioconductor and AltAnalyze.

Another important feature of MAAMD is that it can identify and download the required library files and annotation files automatically. This feature was inherited from its embedded tool, AltAnalyze. There are many microarray platforms which have different probeset libraries. Thus, matched library and annotation files are required to analyze a microarray dataset when starting with raw data files. It takes time for the users to find the proper resources, to understand the library and annotation files, and to interpret the raw microarray data with the matched library and annotation files. MAAMD automatically downloads library files and generates results with gene symbol and pathway information which can be easily understood. Moreover AltAnalyze is able to automatically download the most current gene ID information, pathway and ontology databses with just a few clicks and supports every species in these analyses for which Affymetrix provides a microarray for.

### Extending MAAMD to other profiling platforms and considerations for meta-analyses

As a Kepler-based workflow, MAAMD can be easily extended or modified in Kepler. For example, MAAMD can be easily separated into several independent workflows which are available in the website http://www.biokepler.org/use_cases/maamd-workflow-standardize-meta-analyses-affymetrix-microarray-data. The workflow “MAAMD-Comparison” separates “DataComparison” module from MAAMD and can run as an independent workflow as long as the analyzed data are ready. The workflow “MAAMD-Download” allows users to download all targeted datasets independently from the data analysis. The workflow “MAAMD-AltAnalyze” can run the data analysis for the downloaded data independenty. These separated workflows allow users to use MAAMD in a more flexible way, as long as the users ensure the correct inputs for each workflow. Users who have programming skills can readily add more modules into the workflow or modify the workflow according to their own requirements.

Currently, MAAMD works for Affymetrix microarray datasets. It can be expanded to support more microarray datasets such as Roche and Illumia as both AltAnalyze and *arrayQualityMetrics* are able to analyze such data platforms. Since AltAnalyze supports the meta-analyses of aligned junction and exon sequences, this makes it possible to extend the workflow for the meta-analyses of RNA sequencing data, integrating it with the proper pre-processing tools for sequencing data. However, due to the many differences in analysis steps between microarray data and sequencing data, significant additional modifications would be required to extend MAAMD for sequencing data, e.g. base-level quality control, adapter trimming, alignment, alignment quality control and exon bed file generation.

There are many important caveats that must be considered when drawing conclusions from meta-analyses and inter-dataset comparisons that can compound false-discovery rates [20]. A number of factors to consider are biologically related (e.g. intra-group variability), technically related (platform specific biases, sample preparation biases, missing probe IDs ect…) and those that arise from increasing the numbers of repeated measures and sample sizes. While it is impossible to correct for many of these, we have designed the workflow in such a way to enable the user to at least be aware of potential issues related to some of these. The quality control measures were designed to flag issues with samples that would cause major intra- and inter-group variability. AltAnalyze can also conduct intra-dataset COMBAT analysis, which employs a both parametric and non-parametric empirical Bayes frameworks to remove batch effects [21]. Missing values for orthologues can be expected when conducting meta-analyses and approaches for imputing these values, if necessary, could be explored [22]. Moreover, one must always be wary of drawing extensive conclusions from experiments that do not contain experimental replicates, however there are strategies for dealing with these issues [23]. One advantage of our approach is that MAAMD defines differentially expressed genes within each experiment first before making inter-dataset comparisons. In our experience this serves to control well for many technically related biases such as biases introduced by array type and sample preparation.

## Conclusions

MAAMD standardizes, simplifies, and dramatically decreases the amount of time required to analyze Affymetrix microarray datasets and to make intra- and inter-dataset comparisons. The minimized computer skills and bioinformatics knowledge required to run MAAMD makes it an attractive tool for biologists with limited programming skills and bioinformatics background. The extensibility of MAAMD means it could be a solid starting point for those researchers who have advanced programming skills and want to expand and/or modify the workflow for additional purposes.

## Availability and requirements

**Home Page:**http://www.biokepler.org/use_cases/maamd-workflow-standardize-meta-analyses-affymetrix-microarray-data.

**Operating system:** Windows and Mac OSX.

**Requirements:** R and AltAnalyze are required. The installation instructions and application instance are available in above website.

**Other requirements:** Internet connection.

**License:** Free for non-commercial and academic use.

## Competing interests

The authors declare that they have no competing interests.

## Authors’ contributions

ZG, AZ, AM and IA contributed to the design of the workflow. ZG contributed to workflow implementation. ZG, AZ, AM contributed to the design of study case. JW developed Kepler components and NS updated AltAnalyze to support MAAMD workflow. All authors contributed to manuscript preparation and read and approved the final manuscript.
